# The Effectiveness of Tuberculosis Education Programme in Kelantan, Malaysia on Knowledge, Attitude, Practice and Stigma Towards Tuberculosis among Adolescents

**DOI:** 10.21315/mjms2020.27.6.10

**Published:** 2020-12-29

**Authors:** Nur Aiza Idris, Rosnani Zakaria, Rosediani Muhamad, Nik Rosmawati Nik Husain, Azlina Ishak, Wan Mohd Zahiruddin Wan Mohammad

**Affiliations:** 1 Department of Family Medicine, School of Medical Sciences, Universiti Sains Malaysia, Kelantan, Malaysia; 2 Faculty of Medicine, Universiti Sultan Zainal Abidin, Terengganu, Malaysia; 3 Department of Community Medicine, School of Medical Sciences, Universiti Sains Malaysia, Kelantan, Malaysia

**Keywords:** tuberculosis, adolescents, knowledge, attitude, practice, stigma

## Abstract

**Background:**

Tuberculosis (TB) is contagious and the transmission risk is high in congregate settings like school. Incidence of TB among adolescents is significantly high hence an education programme was developed to improve knowledge, attitude, practice and stigma (KAPS) among them.

**Methods:**

This school-based, non-randomised controlled study was conducted among secondary school students with a total of 236 respondents. The KAPS score were assessed before and 1 month after using self-administered validated KAPS questionnaire on TB. Analysis was done using repeated measures ANOVA.

**Results:**

The mean percentage score (SD) for baseline knowledge, attitude, practice and stigma score for the respondents were 54.0 (4.48), 65.6 (1.74), 70.0 (1.43) and 66.0 (6.88), respectively. There was a significant difference (*P* < 0.001) in the knowledge and stigma score for intervention group compared to control group, adjusted for gender, ethnicity and smoking status 4 weeks post-TB educational programme. However, with regards to attitude and practice score, there was no significant difference (*P* = 0.210 and *P* = 0.243, respectively).

**Conclusion:**

TB education programme was effective in improving knowledge and stigma related to TB. This health education programme can be used as one of the strategies for the prevention and control of TB in schools.

## Introduction

Despite having a comprehensive tuberculosis (TB) control programme, Malaysia has a high number of cases with TB continues to be a global health problem that affects millions of people each year. In 2017, the World Health Organization (WHO) (1) reported the estimated TB rate in Malaysia as 93 in 100,000. Thus, Malaysia was categorised as an intermediate TB-burden country. Malaysia has implemented high quality TB management in combating TB since 1961, yet our treatment success rate for TB remains below 90% (2).

A national study found that children and adolescents accounted for 8.5% of the TB cases with high TB incidence was reported between age group of 10 to 19 years old (3). A survey conducted in Kelantan obtained similar results: 8.4% of the registered TB cases in 2012–2015 were children and adolescents, where the mean age for TB infection was 15.98 years (4). Current scenario indicates that effective TB control strategies involving adolescents should be established to control TB transmission and aim to end the TB. In the present study, the respondents were secondary school students aged 14 and 16 years old. Thus, it represents the age group of Malaysian adolescents who are at risk for TB.

Studies conducted in Malaysia have found a low awareness and knowledge of TB (5–7). Not only was the knowledge about the disease low, but the level of social stigma was reported high (5, 6). Stigma remains a significant challenge for TB control programmes across the prevention-to-care continuum (8, 9) It can prevent people from getting tested, using care services or changing their behaviour to avoid the spread of the disease (10, 11).

With intervention targeting school children was crucial for TB control and towards TB elimination because the risk of TB transmission is high in congregate settings like school, thus making investigating exposures and treating infected contacts become more challenging (12) given the health concerns related to TB, interventions must be well planned in order to be effective. Besides, adolescents are expected to be more receptive to specific health messages, and more easily comprehend the information and relay it to other household members. This was demonstrated in a systematic review of preventive health education in 11 studies, the researchers concluded that health education in schools could have a positive effect on knowledge, attitudes and preventive behaviours (13).

**Figure 1:**
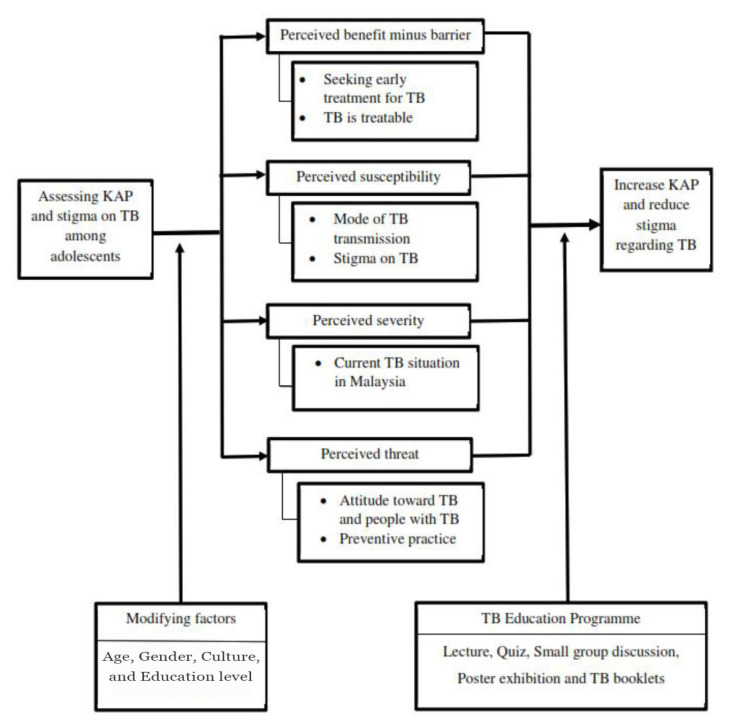
Conceptual framework based on Health Belief Model

The United States Centres for Disease Control (CDC) and Prevention’s Healthy Youth Initiative and the WHO reports have stressed the important role of schools in influencing the health education of future generations (14, 15). The acquisition of health-related knowledge, skills and attitudes can empower children to live healthy lives and to become change agents in their communities. Not only does the provision of health education to children have a short-term effect; it can lay the foundation for their healthy development during adolescence and the rest of their lives. In this study, the Health Belief Model was applied to a TB education programme that aimed mainly to evaluate and increase adolescents’ knowledge, to promote positive attitudes, to encourage preventive behaviours and to reduce stigma as shown in Figure 1, with a programme that increases TB awareness among secondary school children could have a significant effect on prevention and disease control.

## Methods

A school-based interventional study was conducted in two secondary schools in Kelantan between July and November 2017. The sample size was calculated through the comparison of two means between and within the groups with requirement for power 0.80 and assuming a type I error rate of 5%. From this calculation, the four outcome variables i.e. knowledge, attitude, practice and stigma of this study were obtained. Standard deviation of mean difference of stigma within the group was 5.28 (unpublished data) and estimated difference of 1.5, given that the stigma domain yielded the largest sample size (*n* = 98) and was therefore adopted for the study. An additional 20% was chosen to compensate for dropouts; thus, there were 118 respondents in each group. Thus, the total sample size was 236.

A cluster sampling was implemented to select participants, which involved first selecting one school from two districts and allocating the schools to intervention and control groups (non-randomised). The schools were chosen based on their profiles, which included the distance to the nearest city, academic performance and numbers of students, with the participating students from the Pasir Mas district being assigned to the intervention group, and those from the Pasir Puteh district assigned to the control group. In addition, the distance between these two schools, approximately 60 km, avoided contamination effects. The participants were voluntarily consented and literate students in the Second Form (14-year-olds) and Fourth Form (16-year-olds). The recruitment of the respondents started once the approval letter from Human Ethics Committee of Universiti Sains Malaysia, the Ministry of Health and the subsequent permission letter from the Kelantan State Department of Education had been received. The students were chosen subsequent to the receipt of the approval letter from their school principal.

The intervention group received a TB education programme that was developed and validated by a group of experts consisting of a respiratory physician, a family medicine specialist and a public health specialist. The intervention programme was held for a day at the school using multiple education approaches. The programme consisted of a lecture, a quiz session, small group discussions and poster exhibition, and four booklets on TB were given out. A 30-min lecture on overview of TB in general, including common symptoms and benefit of TB treatment was given. A quiz consisting of five questions pertaining to the lecture was conducted afterwards. The session was well received by the students as a correct answer would receive a small token of appreciation (pre-paid top-up with a value of RM5) (including X-ray of TB patients, TB epidemiology, symptoms and clinical presentation) and four booklets (TB symptoms, HIV coinfection, TB contact and TB treatment). Each small-group discussion had 15–20 respondents and was facilitated by a group of trained doctors. The standardisation of these facilitators was done by a researcher team a day before the programme. Two case scenarios (culture-based) were used to address local myth that TB is part of black magic and uncurable with modern medicine and it is a disease of the poor only (16). The case scenarios were also designed to address ignorant attitude and practice towards TB spreads and low knowledge about TB symptoms and treatment in general (unpublished data). Apart from that, we also promoted quit smoking as a preventive measure on reducing the risk of getting TB (17). The duration of the intervention was 4 h.

**Figure 2:**
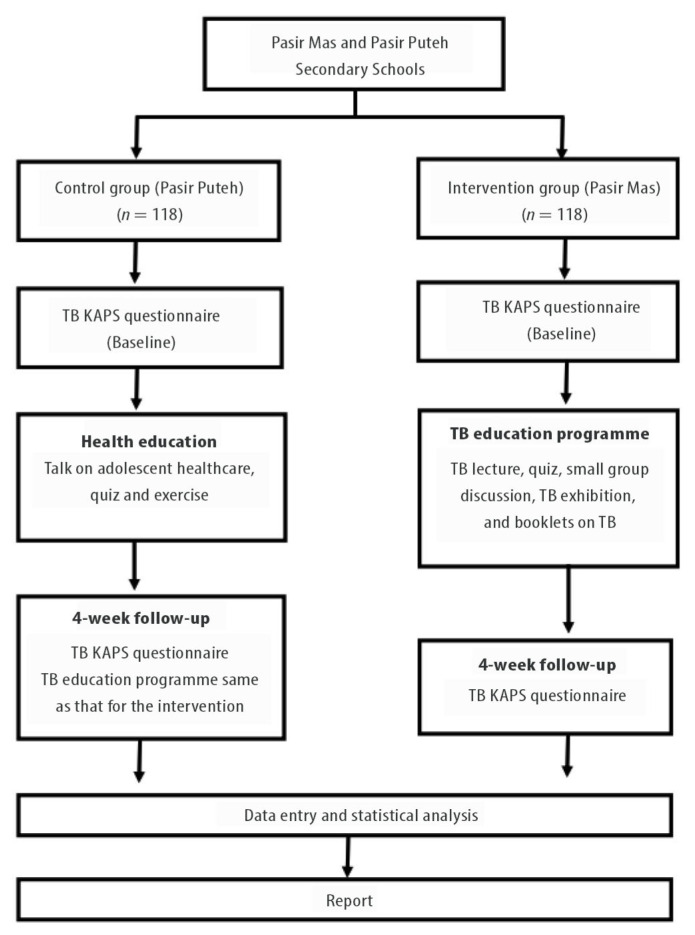
Study flow chart

The control group presented information on adolescent health and hygiene. The intervention evaluation was conducted twice for each group: at baseline and 4 weeks post-intervention. The participant requirements and intervention programmes are presented in Figure 2.

The data was collected through a validated unpublished Malay language version of the TB knowledge, attitude, practice and stigma (KAPS) questionnaire (unpublished data). The questionnaire consisted of five sections: i) socio-demography; ii) knowledge; iii) attitude; iv) preventive practice and v) stigma towards TB. The item for knowledge, attitude and practice domains were constructed from a survey done in 2015 on TB among secondary school students (unpublished data). For the item in stigma domain, it was translated into Malay language from the TB- and HIV/AIDS-related stigma scales by Van Rie et al. (18). The Cronbach’s alpha values for the KAPS domains were 0.621, 0.590, 0.629 and 0.862, respectively.

The first section of the questionnaire had 6 sociodemographic questions relating to age, gender, ethnicity, smoking status, vaping status and usage of substance abuse. The second section concerned knowledge of TB. It contained three subdomains that covered the general understanding of TB (11 items), the symptoms (9 items) and prevention (5 items). Each item was answered with ‘1’ for the correct answer and ‘0’ for the wrong answer or to indicate uncertainty. The maximum score for knowledge was 25. The higher the score, the greater was the students’ knowledge about TB.

The third section measured attitudes to TB and people with the disease (5 items). The items consisted of behaviour and cognitive response towards prevention of TB. The attitude was assessed with a 5-point Likert scale: ‘1’, strongly disagree; ‘2’, disagree; ‘3’, unsure; ‘4’, agree; and ‘5’, strongly agree. The maximum score was 25. The higher the score, the better was the students’ attitude towards TB and those with the disease.

The next section measured preventive practices towards TB (6 items), such as cough etiquette. For each item, the frequency of a practice was indicated by a ‘2’ if it was done almost all the time, ‘1’ if occasionally and ‘0’ if never. The maximum score for the practice section was 12. Thus, the higher the score, the higher was the frequency of the prevention practice.

The last section dealt with stigma towards TB patients (11 items). Stigma was assessed with a 5-point Likert scale: ‘1’, strongly disagree; ‘2’, disagree; ‘3’, unsure; ‘4’, agree; and ‘5’, strongly agree. The maximum score was 55. The higher the score, the greater was the stigma towards TB. All of the scores were reversed for negative statements.

The data were analysed with IBM SPSS Statistics for Windows, version 24.0 software. Descriptive statistics were used for all the variables. Pearson’s Chi-square test and Fisher’s exact test were performed to compare the baseline characteristics of the control and intervention groups. A repeated measures analysis of variance (ANOVA) was used to compare the mean scores within and between the groups. The dependent variables were the KAPS scores with two levels of measurement: at baseline and 4 weeks following the TB education programme. Gender, ethnicity and smoking status were a potential confounder. The level of significance was set at 0.05 with two-tailed fashion.

## Results

A total of 236 secondary school students — 118 in the control group and 118 in the intervention group, comprised the sample. The response rate was 100% in both groups. A majority of the students were Malay. Females predominated. Of the 236 students, 8% indicated that they smoked, 20.3% vaped and a small number, 0.8% abused substances. There were no significant differences in age, vaping status or substance use and abuse between the groups. However, for gender, ethnicity and smoking status, there were statistically significant differences (Table 1).

### Baseline Knowledge, Attitude, Practice and Stigma Score

The mean (SD) pre-intervention knowledge score for the respondents (*n* = 236) was 13.5 (4.48) out of a maximum of 25. The mean (SD) total attitude score was 16.4 (1.74) out of a possible maximum of 25 and the mean (SD) total practice score was 8.4 (1.43) out of a possible maximum of 16. The mean (SD) total stigma score was 36.3 (6.88) out of a possible maximum of 55.

There was no significant difference between the groups for baseline knowledge (*P* = 0.277), practice (*P* = 0.650) or stigma (*P* = 0.086). However, there was a significant difference in the baseline attitude score of the groups (*P* = 0.009).

### Intervention Effects

**Figure 3:**
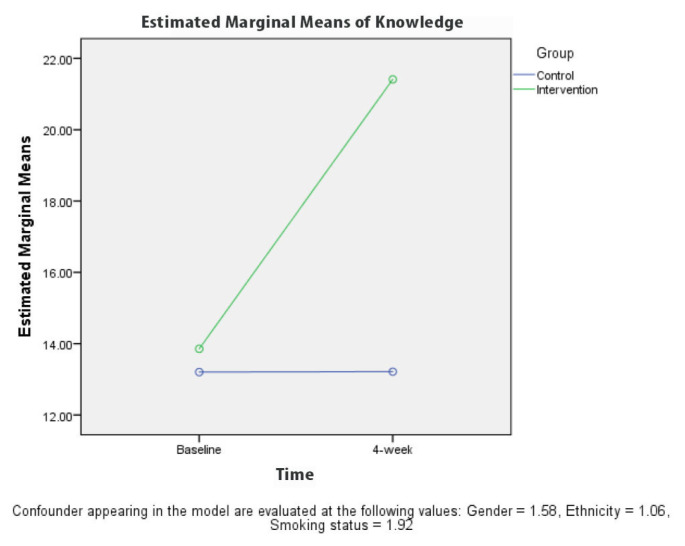
Comparison of mean knowledge regarding TB between each group based on time

**Figure 4:**
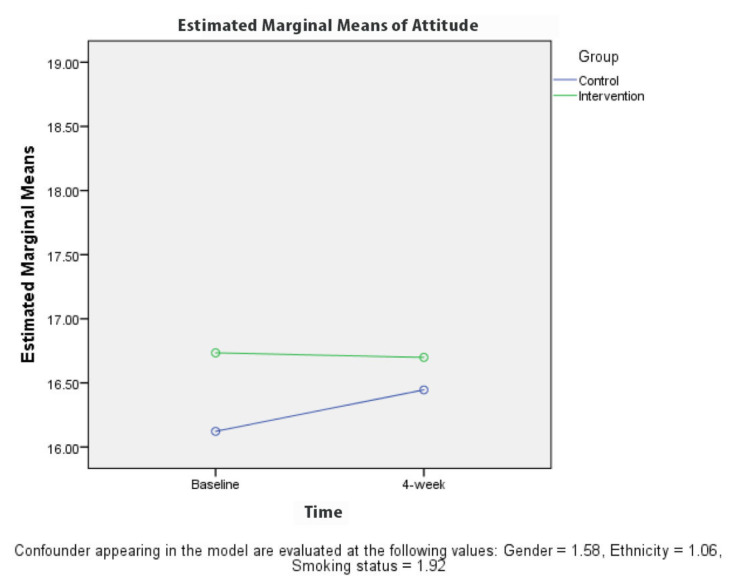
Comparison of mean attitude regarding TB between each group based on time

**Figure 5:**
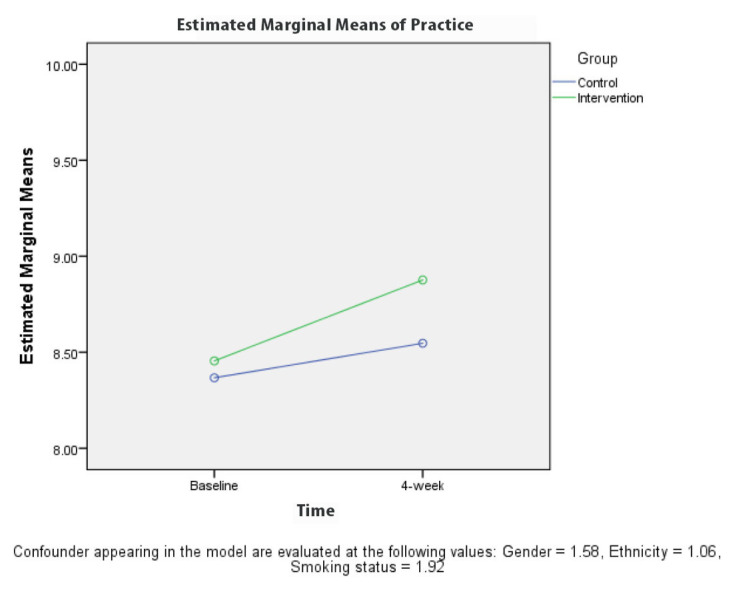
Comparison of mean practice regarding TB between each group based on time

**Figure 6:**
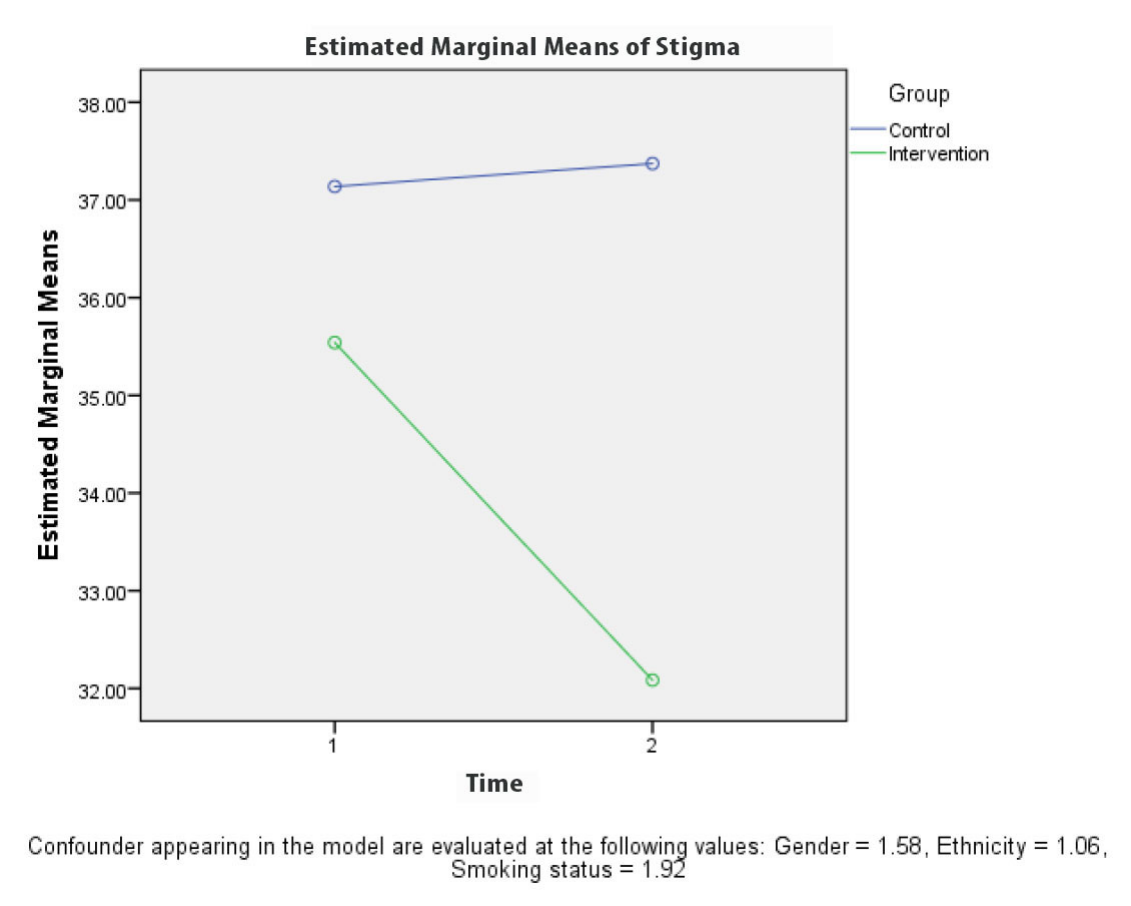
Comparison of mean stigma regarding TB between each group based on time

Table 2 presents the results for the comparison between KAPS scores for the groups at baseline and 4 weeks after the intervention. A repeated measures ANOVA revealed a significant difference (*P* < 0.001) in the knowledge (Figure 3) and stigma (Figure 6) scores for the control and intervention groups, adjusted for gender, ethnicity and smoking status at 4 weeks after the TB education programme. The attitude (Figure 4) and practice (Figure 5) score for the control and intervention groups, adjusted for gender, ethnicity and smoking status, 4 weeks after the education programme revealed that there was no significant difference (*P*-values of 0.218 and 0.243, respectively).

The comparison of the mean KAPS scores on the basis of time and simultaneous group differences (Table 2) revealed a significant improvement in the mean score in all the domains except attitude in the intervention group. In the control group, there was no significant difference in the KAPS score at baseline and 4 weeks after the TB education programme.

## Discussion

This study assessed adolescents’ KAPS towards a TB education programme at school. Where few intervention studies on TB have been conducted worldwide aiming to increase level of knowledge, attitude and practice towards TB, this one-day school-based intervention programme was delivered via a 30-min lecture, quiz, small-group discussions, a poster exhibition and four booklets regarding TB. While to date there has been no specific education intervention programme about TB done among schoolchildren in Malaysia, CDC and WHO had identified that conducting health education in school could promote healthy lifestyle to younger generation and contribute to the overall health to the public (14, 15).

The adolescents in the present study were found to have average levels of knowledge and preventive practices with regards to TB. Overall, they had positive attitudes towards prevention; however, the level of stigma towards the disease was high. This high level of stigma could pose an obstacle to treatment and contact tracing in this group. Lack of knowledge regarding TB symptoms and disease transmission resulted in delay seeking for treatment and increased TB contact.

In the present study, confirming the results of an intervention study conducted in Alexandria, the participants in the TB education programme exhibited a significant increase in knowledge. The intervention study in a health education programme consisted of 90 min lecture-discussion session followed by 30 min questions and answers and, aided by slides and posters provided to 467 secondary school students in 12 schools, the knowledge about modes of transmission, TB symptoms and preventive practice of TB improved significantly (19). Another cross-sectional study was conducted at a Philippines high school with a total population of 1,906 students. A 20-min lecture about TB was presented to the students. The high school students’ knowledge of TB, which was 65.22% at baseline, increased to 86.83% after a health education intervention (20). These findings were similar to those of an intervention study conducted in India. The knowledge levels were significantly improved after a 30-min audiovisual health education session (21). Health education intervention as simple as lectures was proven to improve the knowledge and awareness regarding TB among adolescents. It can be delivered via many approaches and methods. In the present intervention programme, the understanding of TB was increased even 4 weeks post-intervention via a 30-min lecture that included a multimedia presentation, interactive quiz session and poster exhibition. The students also received pamphlets containing information about the disease.

Besides leading to an increase in knowledge, the present intervention programme resulted in a statistically significant improvement in the stigma scores. In addition to the audio-visual session, quiz and printed materials were presented and small-group interactive discussions with a doctor were held. Two case scenarios were created with a focus on stigma, attitude and preventive practices. The interactive session presented a situation to correct the negative perceptions of TB. Few studies have evaluated the effects of health education interventions on TB stigma. Where data on the effectiveness of these strategies are scarce (8), a systematic review of the literature on TB stigma indicated that only a few studies have suggested that TB education programmes aimed at health care professionals, individuals with TB and those at risk might reduce stigma. A focus group study found that individuals enrolled in TB clubs perceived themselves to be less affected by stigma than those receiving standard clinical treatment (22). The clubs provided an environment in which the members’ TB status was highly visible and accepted. In contrast, a quasi-experimental study reported that stigmatising attitudes in the general community in Nigeria had increased after an intervention involving trained community volunteers to develop awareness about TB (23). A reason for the increment of misconceptions could be because of the community volunteers only received 2-day training and not fully understood the cause, transmission, signs, and cure of TB. These findings recommended the need for multiple training sessions with the trainer in future programmes and interventions. In the present study, our intervention activities were handled by trained health care provider, and the respondents’ negative stigma were reduced by giving TB scientific education. Thus by having accurate and adequate knowledge, it was able to reduce the stigma regarding TB.

While several studies showed level of knowledge and awareness was not associated with attitudes and practices, there was no significant change in attitudes and practices over the course of the present educational intervention study. A cross-sectional study involving 250 primary health care centres in Iraq was conducted among 500 patients, found that almost half of the patients had unfavourable attitudes and practices towards TB while 64.4% of them had good levels of knowledge (24). In a multi-centre community cross-sectional study conducted in a population of Saudi Arabia found that most of the respondents had general awareness but not adequate knowledge regarding TB. Majority of them also had negative attitudes towards TB and people with TB. The negative attitude was reported as majority thought they will not suffer from TB, feel fears towards TB and less than half would search for treatment. Of the respondents, 42.3% would avoid people with TB and 29.9% of respondents actually have fear towards them (25). An interventional study done in Iran regarding the effectiveness of TB health education suggested that interventions should focus on the culture and beliefs of a population in order to improve and to maintain positive attitudes. The intervention programme can be led by a trained group or individual consultations concerning their learning and hometown educators with similar beliefs (26). The intervention programme included culturally competent interactive discussion presented through case scenarios that focused on Malay’s perspectives, attitudes and preventive practices towards TB. A majority of our respondents were Malay (94.5%) since the study was conducted in Kelantan, which is located in the northeast of Peninsular Malaysia where the majority of the population is Malay (95.9%) (27). However, the session was held without involvement of their trusted educators to influence their beliefs and culture in a single intervention programme. This element could be the reason for the lack of change in these domains. Culture can affect behaviours through values, beliefs and traditional roles, and, according to a study related to health behaviour among Malaysian adolescents, culture had a great influence on desirable health behaviours among adolescents (28). Healthy behaviour includes good attitude and practices related to health and disease prevention.

Another reason might be the limited period as the study was conducted towards the end of school term, which allowed for only one intervention and evaluation. In a study in Ethiopia conducted to assess the effectiveness of ‘TB clubs’ among TB patients, it was found that this intervention improved societal attitudes towards TB patients and increased patients’ confidence. A weekly meeting to support treatment adherence and to facilitate information sharing had a positive effect on attitudes (22). Repetition and support are essential for promoting positive attitudes and maintaining preventive health behaviours over the long term.

The present study also found an 8% prevalence of cigarette smoking and a 20.3% prevalence of vaping. However, the number of cigarette smokers was comparatively lower than that (11.7%) reported in a 2016 survey of Malaysian adolescents (29). A similar survey also reported that 9.1% of Malaysian adolescents and 7.8% of adolescents in Kelantan were current e-cigarette user (29). A retrospective cross-sectional study of children and adolescents in Kelantan found that cigarette smokers were three times more likely than non-smokers to develop TB infection (17). A case-control study of older children in Brazil also found a relationship between cigarette smoking and TB infection (30). In our education intervention programme, we were able to address this issue to the secondary school students and encourage them to quit smoking during the lecture and small group discussion.

Different methods of health education programme for adolescents have been carried out worldwide and each of the methods had its own limitation and strength, so did in the current health education package. The majority of the respondents were Malays; hence it may not represent the population of Malaysia with multiple ethnic groups. Evaluation for this present study was done using a set of questionnaire, which could lead to bias and inaccurate response. Furthermore, the follow-up period was rather a short period, which was only 4 weeks after the intervention. We were unable to conclude the effectiveness of the education on the KAPS longer than the present duration.

Regardless of the limitations, this did not markedly change the results of the present intervention, which could be due to the appropriate sample size. On the other hand, potential confounders (gender, ethnicity and smoking status) were controlled during analysis to strengthen our study outcomes. The findings of this study can be attributed to the use of the national language for the intervention programme, thus providing better respondents’ perceptiveness.

As the analysis and findings in this study had demonstrated, knowledge alone did not influence adolescent’s attitude and practice. Their belief and culture also had great influence on their health behaviours. While this health education programme could be used as a comprehensive guideline for health professionals or teachers to deliver the message to the students, involvement of teachers or their hometown educators with similar beliefs in giving health education and repetitions of the intervention programme have a say in improving and maintaining positive attitude.

## Conclusion

The role of health education is significant for the dissemination of accurate information and the modification of attitudes and lifestyles. Where the secondary school health education intervention programme in this study was effective for increasing knowledge and reducing stigma but not for improving attitudes and practices, disease awareness will nevertheless facilitate the development of personal health-seeking behaviours and improve perspectives on TB. This TB education intervention could be used as culturally competent intervention and could assist teachers or communities in delivering continuous health education to the adolescents about TB. Intervention for school children was crucial for TB control as the risk of transmission is high in congregate settings like school.

## Figures and Tables

**Table 1:** Respondents’ sociodemographic characteristics (*n* = 236)

Characteristics	Control (*n* = 118) *n* (%)	Intervention (*n* = 118) *n* (%)	*P*-value€
Age group			
14	40 (33.9)	30 (25.4)	0.154
16	78 (66.1)	88 (74.6)	
Gender			
Male	40 (33.9)	58 (49.2)	0.017
Female	78 (66.1)	60 (50.8)	
Ethnicity			
Malay	107 (90.7)	116 (98.3)	0.019*
Non-Malay	11 (9.3)	2 (1.7)	
Smoking status			
Yes	5 (4.2)	14 (11.9)	0.031
No	113 (95.8)	104 (88.1)	
Vaping status			
Yes	11 (9.3)	13 (11.0)	0.667
No	107 (90.7)	105 (89.0)	
Use of substance abuse			
Yes	1 (0.8)	0 (0.0)	0.316
No	117 (99.2)	118 (100.0)	

Notes:

€ Chi-square test;

* Fisher’s exact test; Level of significance was set at 0.05

**Table 2:** Mean difference of KAP and stigma on TB between groups analysis, time-based comparison by using repeated measures ANOVA

Domain	Mean^a^ (SD)		Adjusted mean^b^ (95% CI)		*F* stats (df)	*P*-value^c^
	Intervention	Control	Intervention	Control		
**Knowledge**						
Baseline	13.8 (4.73)	13.3 (4.22)	13.9 (13.03, 14.68)	13.2 (12.38, 14.03)	195.0	< 0.001
4-week	21.4 (3.80)	13.2 (3.96)	21.4 (20.71, 22.12)	13.1 (12.51, 13.92)	(1, 231)	
**Attitude**						
Baseline	16.8 (1.76)	16.1 (1.68)	16.7 (16.42, 17.05)	16.1 (15.81, 16.44)	1.5	0.218
4-week	16.7 (1.71)	16.4 (2.06)	16.7 (16.35, 17.05)	16.4 (16.10, 16.80)	(1, 231)	
**Practice**						
Baseline	8.4 (1.41)	8.4 (1.46)	8.5 (8.19, 8.72)	8.4 (8.10, 8.63)	1.4	0.243
4-week	8.9 (1.61)	8.5 (1.68)	8.9 (8.57, 9.18)	8.5 (8.24, 8.85)	(1, 231)	
**Stigma**						
Baseline	35.6 (7.14)	37.1 (6.54)	35.5 (34.27, 36.81)	37.2 (35.87, 38.41)	12.7	< 0.001
4-week	32.3 (9.52)	37.2 (6.47)	32.1 (30.62, 33.55)	37.4 (35.90, 38.84)	(1, 231)	

Notes:

a Descriptive mean;

b based on estimated marginal mean;

SD = standard deviation; CI = confidence interval;

c Group-time interaction of repeated measure analysis of variance; Adjusted for gender = 1.58, ethnicity = 1.06 and smoking status = 1.92

**Table 3:** Comparison of mean KAPS regarding TB within each group based on time by using repeated measures ANOVA

Comparison	Control		Intervention	
	Mean diff (95% CI)	*P*-value€	Mean diff (95% CI)	*P*-value€
**Knowledge**				
At 4-week baseline	−0.01 (−0.75, 0.73)	0.976	7.56 (6.82, 8.30)	< 0.001
**Attitude**				
At 4-week baseline	0.32 (−0.08, 0.72)	0.111	−0.04 (−0.43, 0.36)	0.861
**Practice**				
At 4-week baseline	0.18 (−0.10, 0.46)	0.212	0.42 (0.14, 0.71)	0.004
**Stigma**				
At 4-week baseline	0.24 (−1.18, 1.65)	0.745	−3.46 (−4.87, −2.04)	< 0.001

Notes:

€ Repeated measures ANOVA; The mean difference is significant at the 0.05 level; Adjustment for multiple comparisons: Bonferroni
